# Preparation of Calcipotriol Emulsion Using Bacterial Exopolysaccharides as Emulsifier for Percutaneous Treatment of Psoriasis Vulgaris

**DOI:** 10.3390/ijms21010077

**Published:** 2019-12-20

**Authors:** Bo Song, Ruiteng Song, Min Cheng, Hairong Chu, Fang Yan, Yuzhen Wang

**Affiliations:** 1 School of Pharmacy, Weifang Medical University, Weifang 261042, Shandong, China; songsbob@163.com (B.S.);; 2 Clinical Medical School, Weifang Medical University, Weifang 261042, Shandong, Chinajingjingllk@163.com (H.C.)

**Keywords:** exopolysaccharides, psoriasis, calcipotriol, emulsion

## Abstract

An exopolysaccharides/calcipotriol (EPS/CPT) emulsion was prepared using bacterial EPS as emulsifier, sunflower oil as an oil phase and CPT as the loaded drug, and the effect of this emulsion on psoriasis vulgaris treatment was evaluated. An EPS composed of mannose (70.56%) and glucose (29.44%) was obtained from the marine mangrove bacteria *Bacillus amyloliquefaciens* ZWJ (Zhu Wenjing) strain. The EPS has significant emulsifying activity at the concentration of 1.5%. The prepared EPS/CPT emulsion has small and stable particle size, with a drug content of 0.00492%, and good spreading properties. The in vitro drug release results revealed that the emulsion showed a certain sustained release effect. In vitro and in vivo animal experiments show that the EPS/CPT emulsion can effectively treat psoriasis vulgaris by increasing the accumulation of CPT in psoriatic skin lesions and reducing the levels of inflammatory cells and inflammatory factors (TNF and IL6). Additionally, it has a certain effect on reducing the side effects associated with CPT. This study lays a foundation for the research of EPS in the topical application of medical materials and treatment of psoriasis.

## 1. Introduction

The treatment and prevention of psoriasis is currently one of the major research topics in dermatology. About 120 million people worldwide suffer from psoriasis, 80–90% of whom suffer from psoriasis vulgaris, which often affects the scalp, face, back, elbow and knee [1]. It is generally believed that abnormal proliferation and differentiation of keratinocytes (KCs) in psoriatic lesions is a key aspect in the pathogenesis. Therefore, the use of topical agents to inhibit KC cell proliferation and differentiation is an important means for the treatment of psoriasis vulgaris [2].

Calcipotriol (CPT) is a first-line topical drug for the clinical treatment of psoriasis vulgaris. It is a synthetic derivative of calcitriol, the active form of vitamin D, which can specifically bind to the vitamin D receptor to exert a gene regulatory effect leading to the attenuation of the abnormal proliferation and differentiation of KCs. Additionally, CPT inhibits the inflammatory response and reduces the number of T cells in psoriatic lesions [3]. Currently, there are mainly external dosage forms, such as creams and tinctures, which are low cost and convenient to use. However, long-term application of CPT increases the loss of moisture in the epidermis, and causes side effects, such as skin atrophy, light sensitivity, irritation and local allergies. In order to avoid these side effects, topical preparations with moisturizing properties are often used in combination with long-term use in the clinical treatment of psoriasis [4].

An emulsion, with its low surface tension, is a better form for transdermal administration. It is easy to spread and moisturize the skin when percutaneously administered, affecting the skin structure and promoting drug penetration, thus its use in the treatment of local inflammation, psoriasis or specific dermatitis and other skin diseases is very suitable [5]. For example, a eucalyptus oil emulsion prepared with Tween 20 and ethanol as an emulsifier can improve skin and drug interactions and safely and effectively treat psoriasis percutaneously [6]. Currently, commonly used emulsifiers include Span 80, Tween 80 or phospholipids, with few choices. At present, researchers are making great efforts to find novel and safe emulsifiers with stronger emulsifying activity [7].

Exopolysaccharides (EPS) have significant moisturizing properties. EPS are natural long-chain polymers. They can hold several times or even dozens of times more water, so are often used as a skin moisturizer. In addition, the significant emulsifying activity and emulsion stability of EPS are also among their most important physicochemical properties. Its spatial stability creates an extended network in the continuous phase to stabilize the emulsion [8]. For decades, many EPS have been discovered by researchers from a variety of strains and have shown significant emulsifying and stabilization properties of the emulsion at relatively low concentrations (around 1.0%). In summary, EPS have great potential for applications in pharmaceutical materials, such as humectants and emulsifiers [9]. The use of some EPS has been reported in the treatment of psoriasis. For example, swimming in the Icelandic blue lagoon has a good therapeutic effect on psoriasis. The EPS produced by the main strain of *Cyanobacterium aponinum* in this blue lake water can increase IL10 secretion from dendritic cells and reduce IL17 release from T cells, which may be responsible for the beneficial effects of the blue lake water in the treatment of psoriasis [10]. However, to our knowledge, studies on the treatment of psoriasis with EPS as a moisturizer and drug-loading material have not been reported to date.

In this study, a marine mangrove system was screened for EPS-producing bacteria and a strain of *Bacillus amyloliquefaciens* (named ZWJ strain) was identified as a producer of high-emulsification EPS and its EPS production process was evaluated. After the EPS was extracted and separated, the physicochemical properties, such as molecular weight (Mw), monosaccharide composition, moisturizing and emulsification were studied. Given its superior emulsifying properties, it was prepared into an emulsion and encapsulated with CPT drug, and the particle size, type, drug loading, stability and drug release in vitro of the EPS/CPT emulsion were assessed. Ultimately, the effects of the EPS/CPT emulsion on psoriasis vulgaris were evaluated by animal experiments, which provided a scientific basis for the application of EPS as a moisturizer and emulsifier in the field of pharmaceutical materials.

## 2. Results

### 2.1. Strain Identification and Growth Curve

**Figure 1:**
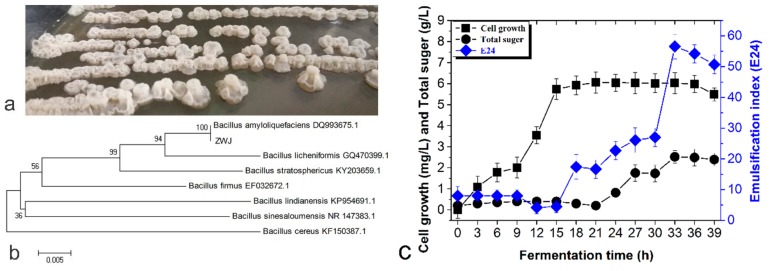
(**a**) The morphology of the colonies; (**b**) Consensus tree; (**c**) Time course of cell growth, total sugar and E during the fermentation of the ZWJ (Zhu Wejing) strain.

The ZWJ strain produced white colonies with high water content on the plate YPD medium, with intermediate and marginal irregular protrusions (Figure 1a). The 16S rDNA gene sequence of this strain (Genbank accession number: MG 575898) was compared and the phylogenetic tree was constructed (Figure 1b) to identify the interspecific relationship and classification of the strain. The ZWJ strain and *B. amyloliquefaciens* DQ993675.1 segregated into a cluster, and their high homology and close evolutionary distance indicate that ZWJ strain belongs to the *B. amyloliquefaciens* species.

In order to determine the optimum fermentation time for EPS production, the E and polysaccharide content of the supernatant of the fermentation broth at different fermentation time points were monitored. The growth curve (Figure 1c) reveals that the polysaccharide content and emulsifying activity are at the highest level at 33 h. Thus, 33 h was ultimately selected as the fermentation termination time, and the extraction rate of EPS was about 0.45 g/L.

### 2.2. EPS Preparation, Mw Determination and Monosaccharide Analysis

The results of the isolation and purification process, Mw, monosaccharide composition, HPLC and ^1^H-NMR analyses of the EPS are shown in the Supplementary Materials. When the fermentation medium of the strain was glucose:peptone:yeast extract (1:2:2), the produced EPS was composed of mannose and glucose (70.56%:29.44%), and the Mw was 2.61 × 10^5^ Da.

### 2.3. Emulsifying Behavior of the EPS

**Figure 2:**
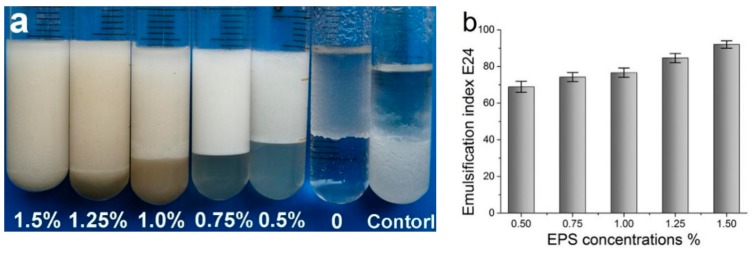

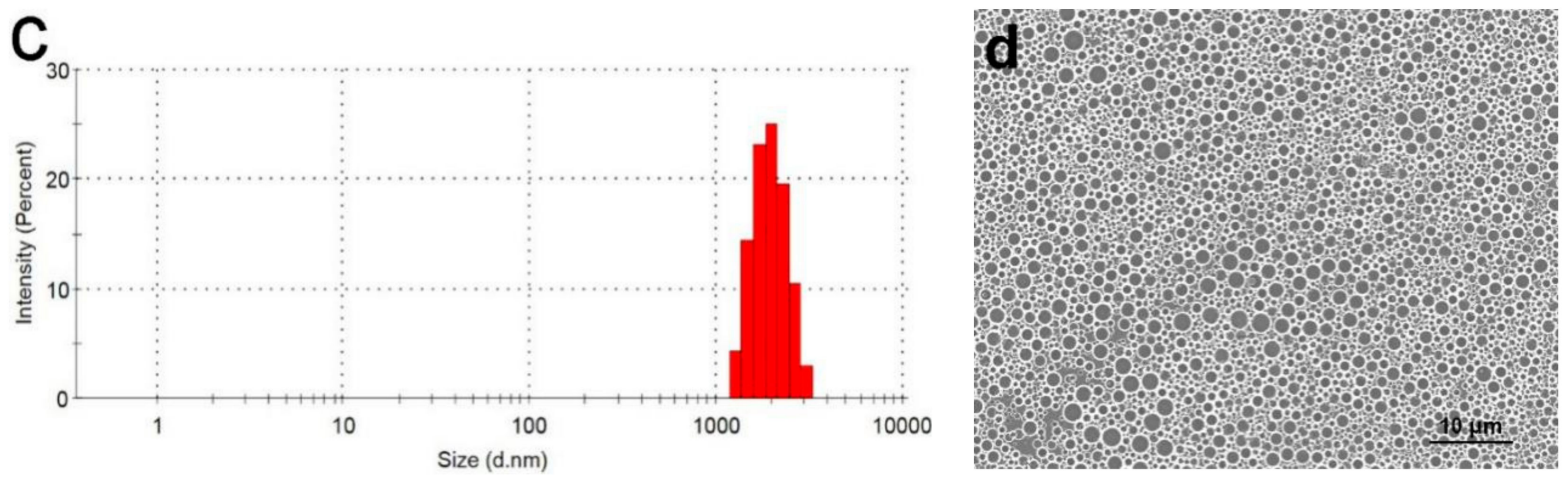
(**a**) Exopolysaccharides (EPS) emulsified sunflower oil at different concentrations; (**b**) different EPS concentrations on the stability of the emulsion; (**c**) droplet size distribution (droplet size = 2357 nm); (**d**) Microscopic morphology of the emulsion droplets.

The emulsification indices (E_24_ and E_168_) of EPS for four vegetable oils are listed in Table S4 in the Supplementary Materals. Among them, the EPS had the highest emulsifying activity on sunflower oil, and the EPS was significantly better than the control group with xanthan gum (*p* < 0.05). In addition, the data in Figure 2a,b show that E_24_ increased gradually when the EPS concentration increased from 0.5% to 1.5% (*p* < 0.05). Moreover, when the morphology of each emulsion droplet was observed under an inverted microscope, the droplet size of the EPS emulsion was small and stable, and the droplet size distribution was also very narrow (Figure 2c,d). According to the above results, when sunflower oil was selected as the oil phase and 1.5% EPS was used as the optimum concentration, a relatively stable emulsion was obtained, and the droplet size was smaller and uniform.

### 2.4. Preparation and Physicochemical Characteristics of the EPS/CPT Emulsion

The physicochemical properties of the prepared EPS/CPT emulsion, such as emulsion type, viscosity, spreadability, droplet size and storage stability, are described in the Supplementary Materials. The summary of the results listed in Table 1 reveals that the viscosity of EPS/CPT emulsion is moderate.

### 2.5. Drug Release from the EPS/CPT Emulsion In Vitro

**Figure 3:**
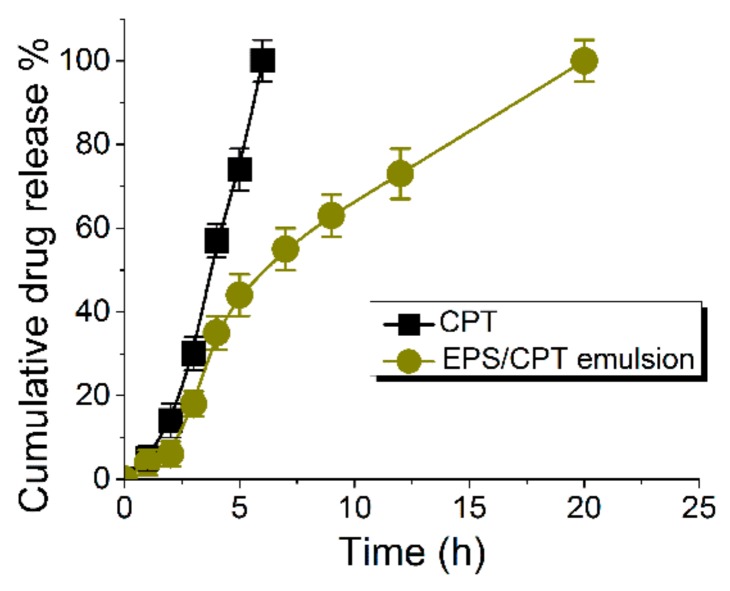
In vitro drug release profile of EPS/CPT emulsion and free calcipotriol (CPT).

In vitro drug release analysis (Figure 3) showed that the cumulative release of free CPT at 6 h was 95.1 ± 5%, and the cumulative release from the EPS/CPT emulsion at 20 h was 93.3 ± 4%. The release time of EPS/CPT emulsion was 2.33 times longer than that of free CPT, indicating a certain sustained release effect. The EPS itself has a certain viscosity, and the prepared EPS/CPT emulsion also has a high viscosity. A certain viscosity allows the drug to be uniformly and stably dispersed throughout the system, thereby allowing the drug to diffuse more slowly into the medium for sustained release.

### 2.6. Skin Permeation and Dermal Pharmacokinetics

**Figure 4:**
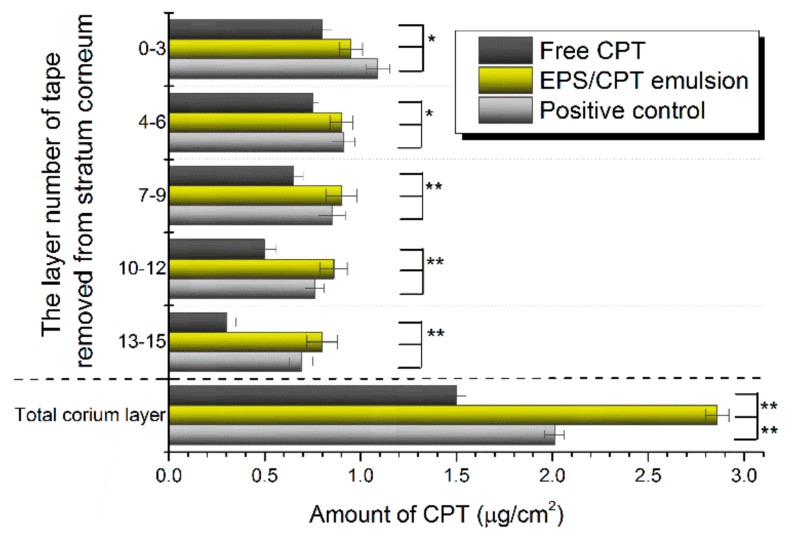
Distribution of CPT from EPS/CPT emulsion, free CPT and positive control (Daivonex) in the skin (Above the imaginary line is depth of penetration of CPT in the stratum corneum; below the imaginary line is amount of CPT in the corium layer; * *p* < 0.05, ** *p* < 0.01).

By HPLC analysis, no CPT was detected in the receiving solution collected at any time point from every group, indicating that CPT did not penetrate the dermis. The results of the experiments with the tape stripping method (Figure 4, dotted above) showed that in the stratum corneum, the total drug content of the free CPT group was 3.0 μg/cm^2^, and the drug content in layers 7–15 was significantly reduced. The total drug content in the stratum corneum of the EPS/CPT emulsion group was 4.41 μg/cm^2^, and the drug content was higher in each layer of the 1–15 layers, which was comparable to the positive control (4.30 μg/cm^2^).

Also, after CPT penetrates the stratum corneum, the drug accumulation in the dermis layer gradually increases, and the drug content in the dermis layer is significantly higher than that in the stratum corneum (Figure 4, under the dotted line). In the dermis layer, the drug content of the EPS/CPT emulsion group was 1.91 times that of the free CPT group, and 1.40 times that of the positive control, indicating that the EPS/CPT emulsion has a stronger penetrating ability. The accumulation of the drug in the affected area of psoriasis increases the chances of drug contact with KC and inflammatory cells.

### 2.7. Psoriasis Animal Experiments

#### 2.7.1. Histomorphology and Inflammatory Factors

**Figure 5:**
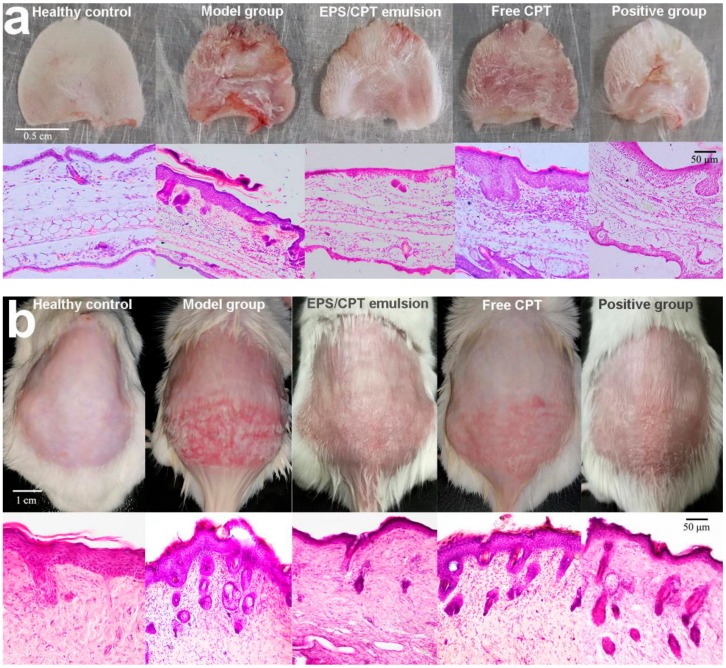

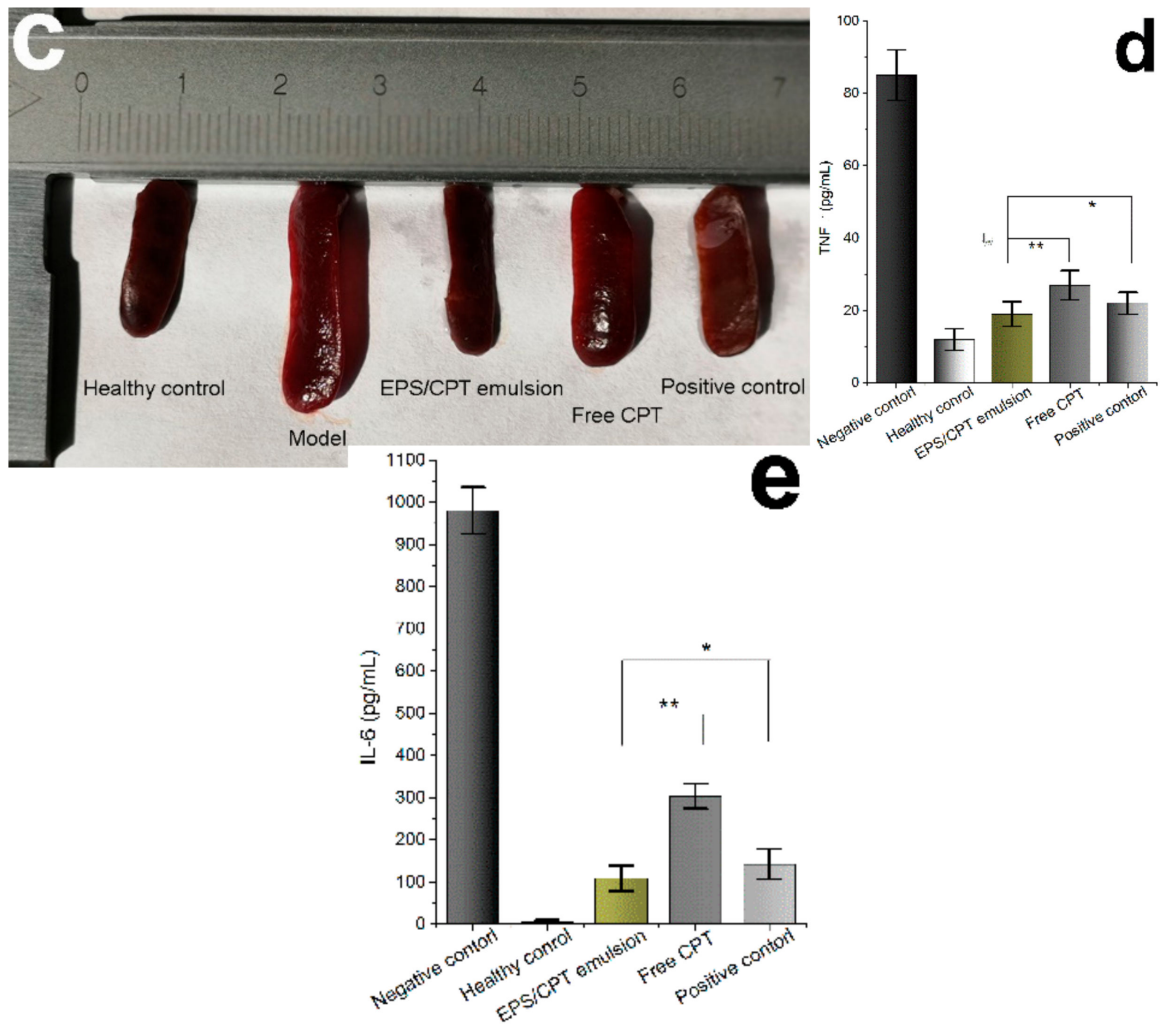
(**a**) Changes in the appearance of the right ear and (**b**) back skin after 7 days of treatment in each group animals and hematoxylin and eosin (H&E) staining images of ear and skin for different groups (200×); (**c**) Changes in spleen size; (**d**) TNF and (**e**) IL6 levels in blood serum from different animals (* *p* < 0.05, ** *p* < 0.01).

Between the first and eighth day of modeling, the psoriatic-like inflammation in the back and right ear of the mice gradually increased. As shown in the healthy control group in Figure 5a,b, the right ears and skin on the back of the mice were intact and there were no symptoms of psoriasis such as scales, erythema and redness. After modeling (as shown in the model group Figure 5a,b), the skin is damaged, red and swollen and the psoriatic-like lesion is more serious in the untreated groups.

The drug was administered daily from the 9th day, and the psoriasis area and severity index (PASI) scores were assessed and are shown in Table 2. On the third day of treatment, the symptoms of the mice in the EPS/CPT emulsion group were significantly reduced compared with those of the free CPT group, which were similar to those of the positive control group. On the 7th day of treatment, in the EPS/CPT emulsion group the redness and psoriatic-like lesion had disappeared, the scales were significantly reduced, some hair recovery had occurred, and the treatment effect was better than that of the free CPT and the positive control group (Figure 5a,b).

Skin histology by hematoxylin and eosin (H&E) staining (Figure 5a,b) also showed that the healthy control group had intact skin, thin stratum corneum and complete keratinization, and no inflammatory cell infiltration. In the model group, epidermal hyperplasia was observed in the psoriatic lesions, and the epidermal hyperplasia was drumstick-like, with a large number of lymphocytes, infiltration of other inflammatory cells and neutrophil aggregation. Symptoms such as parakeratosis and inflammatory cell infiltration were significantly alleviated after treatment. In addition, the order of spleen size of each group was as follows: model group spleen > positive control group > free CPT > EPS/CPT emulsion > normal group (Figure 5c).

The changes in blood serum TNF and IL6 levels in each experimental group are shown in Figure 5d,e, respectively. In the model group, the levels of TNF and IL6 were as high as 85.5 ± 7.0 and 980.1 ± 62.0 pg/mL, respectively, which again demonstrated that the modeling of the experiment was successful. After administration of the EPS/CPT emulsion, the levels of TNF and IL6 in the treated group were significantly reduced compared with other groups. Compared with the model group, the two cytokines decreased by 77.6% and 88.9%, respectively. There was a certain difference from the positive control group (* *p* < 0.05), which was significantly different from free CPT (** *p* < 0.01). The above data indicate that the treatment effect of the EPS/CPT emulsion group is more effective.

#### 2.7.2. Evaluation of Skin Irritation

The scoring results, shown in Table 3, are in the following order: Capsaicin > Daivonex > EPS/CPT emulsion, which clearly indicate that the EPS/CPT emulsion is less irritating than the control Daivonex.

## 3. Discussion

*Bacillus amyloliquefaciens* produces polysaccharides, proteins (including enzymes), and peptides during the growth process, and the composition of the fermentation broth is more complex [11]. In particular, hydrolase, such as cellulases may hydrolyze polysaccharides and affect polysaccharide yield. Therefore, the characterization of the production process of the EPS by the ZWJ strain has certain guiding significance for determining the optimal fermentation time and increasing the yield of polysaccharide.

Many studies have shown that, even in the same strain of bacteria, the produced EPS under different environments, culture media and purification conditions will be different. For example, in the *B. amyloliquefaciens* sc-1 strain isolated from ready-to-eat sliced apple grown in Luria–Bertani (LB) medium with 1.0% glucose (pH = 7.5) as fermentation medium, two EPSs composed of glucose: mannose: galactose: arabinose (15:4:2:1) and glucose: mannose (3:1) were obtained, and the Mw were 0.796 × 10^5^ Da and 1.98 × 10^5^ Da, respectively [12]. The *B. amyloliquefaciens* lpl061 strain with 22.0 g/L sucrose and 18.4 g/L yeast extract (pH = 7.0) as fermentation medium, was cultured at 28 °C for 24 h, separated and extracted to obtain EPS1 (mannose/glucose = 96.9/3.1, 2.33 × 10^5^ Da) and EPS2 (mannose/glucose = 65.3/34.7, 0.981 × 10^5^ Da), and exhibited significant emulsification activity at 1.0% [13]. Different from the previous reports, the *B. amyloliquefaciens* ZWJ strain in this study was derived from the marine mangrove system. The literature reports and the results of this experiment verify that the EPS from *B. amyloliquefaciens* is composed of mannose and glucose, and the Mw is between 10 × 10^5^–30 × 10^5^ Da, but the ratio of the two monosaccharides may vary with the medium and culture conditions.

In order to screen for the optimum oil phase and optimum concentration for the preparation of the emulsion, the emulsification behavior of the EPS was investigated. The droplet size of the emulsion droplets is very important for its physical stability and emulsification rate. The smaller the droplet size, the slower the emulsification rate and thus the emulsion is more stable [14]. The results with the selected oil phase of the sunflower oil in this experiment are consistent with those reported by Prasanna [15] and Han [13]. Another similar study also showed that the emulsification of sunflower oil was higher than that of mustard oil, soybean oil, castor oil and corn oil with EPS (1.0%) produced by *B. coagulans* RK-02 as emulsifier [16]. Iyer [17] reported that the EPS (1.0%) produced by *Enterobacter cloaceae* had the best emulsification activity for peanut oil. Vegetable oil is mainly composed of saturated and unsaturated fatty acids, and is non-toxic and non-irritating. As an oil phase, it has the advantages of good lubricity, high viscosity temperature index and good viscosity-temperature performance, which can improve the stability of the emulsion when stored at low temperature. Sunflower oil is rich in sterols, vitamins and linoleic acid, which can promote the regeneration of human cells and protect skin health. Thus, it is very suitable for the treatment of skin diseases.

The viscosity of the emulsion is largely determined by the emulsifier. Viscosity is the main influencing factor in emulsion spreadability, and the two are inversely related. A moderate viscosity is favorable to improving the coating performance and good skin feel of the emulsion [18,19]. In addition, a certain viscosity will help the adhesion of the emulsion to the surface of the skin, as well as the release and absorption of the drug. The droplet size distribution data show that the droplets are relatively uniform and concentrated, and the uniform droplet size distribution can result in a more uniform drug release rate and avoid the drug burst phenomenon. The analysis of the drug content indicates that the CPT drug content in the emulsion is within the effective dose range and has a guiding effect on the weekly dose and the maximum tolerated dose. The results of the storage stability revealed that although the droplet size slowly increased, the emulsion remained white milky in color, with no delamination and no aggregation, indicating the high storage stability of the EPS/CPT emulsion. EPS can form a solidified film between the oil phase and the water phase to stabilize the emulsion and improve the stability of the emulsion, which may be the main factor determining the EPS emulsion stability [8].

It is well known that long-term use of CPT has a certain irritating effect on the skin, and there is a risk of developing hypercalcemia. Therefore, clinically, there is a certain control on the dosage of CPT drugs, which recommends use only 1–2 times a day, and a maximum dose per week that should not exceed 500 mg. In conclusion, sustained release of CPT appears to be beneficial in the treatment of psoriasis as it leads to its adequate absorption, which helps to reduce drug toxicity [20]. In similar studies, Kaur [4] prepared a clobitasol propionate and CPT nanoemulsion that completely released CPT at 10 h. Also, the cumulative release of CPT from betamethasone dipropionate and CPT loaded solid lipid nanoparticles (CT-BD-SLNs) was only 31.0% at 48 h [21].

In order to further study the permeability and pharmacokinetics of CPT in isolated skin, a thicker, less permeable pig ear skin was used in this study, instead of thickened psoriasis skin. The formation of scaly plaques in the psoriasis area and the thickening of the stratum corneum cause the epidermis to become rough and become a major obstacle to the transdermal absorption of topical drugs [21]. The ability of free CPT to pass through psoriasis skin is primarily affected by the difference in drug concentration. While the EPS/CPT emulsion is affected by the difference in concentration, this emulsion also has the advantages of sustained release, larger specific surface area, higher dispersion and lower surface tension. Such advantages have played a positive role in both drug penetration and therapeutic effects. In conclusion, together, the drug release, percutaneous penetration and pharmacokinetic results indicate that the EPS/CPT emulsion not only has a sustained release effect to prolong the duration of drug action, but also promotes the penetration of CPT and the amount accumulated in the dermis. This is very beneficial for reducing the dose of medications, increasing bioavailability and reducing drug toxicity.

The method used in this study to induce psoriasis-like symptoms in mice using Imiquimod produced successful results. Psoriasis is an immune inflammatory disease, the pathogenesis of which involves a variety of immune cells, such as neutrophils, dendritic cells, and CD4+ T cells. Such immune cells interact with KCs and participate in immune activation to amplify the inflammatory response by promoting the secretion of large amounts of IL6, TNF, and other pro-inflammatory factors, such as IL17 promote KC proliferation and differentiation [22]. Macrophages and lymphocytes (T and B cells) in the spleen perform immune functions, start to produce a large amount of immune substances, such as immunoglobulins and complements, and exert immunological effects, in addition to the enlargement of the spleen. When the levels of inflammatory factors are reduced, the spleen can return to a normal physiological status [23].

The experimental results of this study show that the EPS/CPT emulsion can effectively reduce the levels of the inflammatory factors IL6 and TNF in peripheral blood to alleviate the symptoms of psoriasis. TNF is a proinflammatory cytokine produced by KC. The abnormal proliferation and differentiation of KCs are regulated by many factors, such as IL6 and TNF, which affect the activity, function and proliferation of inflammatory cells (especially T cells) in skin lesions [22]. Increased IL6 levels is one of the conditions for inducing IL23 production by mouse and human Th17 cells [24]. Blocking the release of IL6 from the epidermis of psoriasis patients inhibits IL23-induced IL17 production [25]. The external use of CPT can inhibit the TNF-induced IL6 production by stimulated KCs, thereby leading to the inhibition of KC hyperproliferation and induction of KC differentiation, ultimately reducing inflammation. In a recent similar study, the prepared CPT nanoemulsions inhibited TNF and IL6 by 77.77% and 95.00%, respectively [4].

In summary, animal experiments in this study showed that the EPS/CPT emulsion is more effective in treating psoriasis. This may depend on the physicochemical properties of the EPS/CPT emulsion, such as sustained CPT release by the EPS/CPT emulsion itself, excellent spreadability, low surface tension, large dispersion and specific surface area, which result in a more uniformly distributed drug, with longer acting time and higher dermal accumulation. Moreover, in addition to the potential antioxidant capacity, good water absorption and moisturizing properties of EPS, both its biocompatibility and bio-adhesiveness are also advantages that cannot overlooked.

To investigate the safety of the EPS/CPT emulsion, skin irritation experiments were conducted. The formula of the marketed drug Daivonex contains about 45% propylene glycol to improve the solubility of CPT and the moisturizing property of the product. However, long-term use of high concentrations of propylene glycol will have a cumulative effect, which has a certain impact on the structure of the epidermis and sebum, and produces a burning sensation, a tingling sensation or an itchiness. It is also noteworthy that the CPT drug itself will increase the moisture loss of the epidermis and cause some irritation, which may be the reason for the slight erythema on the 4th day of EPS/CPT emulsion.

The safety exhibited by the EPS/CPT emulsion may be related to the moisturizing properties of EPS (Figure S5) and the safe penetration mechanism. The moisturizing properties of EPS can reduce the adverse effects of CPT on the skin by restoring the permeability, viscosity and water retention capacity of skin cells interstitial [5,26]. The combination of a humectant and CPT reduces inflammation and dryness in the skin lesions, softens the skin, relieves itching and pain, and is beneficial for long-term treatment [4]. It has been reported that EPS can hydrate the stratum corneum of the skin, thereby changing the stratum corneum of the epidermis and enlarging the pores to increase the percutaneous penetration of the drug. This mechanism for the enhancement of its penetration is relatively safer than the use of a penetration enhancer, such as dimethyl sulfoxide [27]. EPS is a natural non-toxic and non-irritating polysaccharide with good biocompatibility. This is very beneficial for product safety, patient compliance and long-term treatment. In summary, this study shows the application prospect of EPS as a medical material in the treatment of skin diseases.

## 4. Materials and Methods

### 4.1. Strain and Reagents

The components of the fermentation medium for the ZWJ strain included glucose (5.0 g/L), peptone (10.0 g/L), and yeast extract (10.0 g/L), pH = 6.5–7.0, and the preservation temperature was –80 °C. Sunflower oil, corn oil, peanut oil and soybean oil were purchased from Shandong Luhua Group Co., Ltd. (Jinan, China) and the date of their manufacturing was within 6 months from the date of the experiment. CPT was purchased from Shanghai Macklin^®^ Reagent Co., Ltd. (Shanghai, China). Imiquimod cream was obtained from Sichuan Mingxin Pharmaceutical Co., Ltd. (Chengdu, China). The enzyme-linked immunosorbent assay (ELISA) kits for TNF and IL6 (Sigma-Aldrich Co., Saint Louis, MO, USA). Positive control drug was Daivonex (CPT scalp solution; LEO Pharma A/S, Ballerup, Copenhagen, Denmark).

### 4.2. Strain Identification

The ZWJ strain was identified through morphological and molecular analysis [28], after amplification of the extracted 16S rDNA gene with the following positive- and negative-strand primers: 5′-CAGAGTTTGATCCTGGCT-3′ and 5′-AGGAGGTGATCCAGCCGCA-3′, using the BLAST alignment tool available in the NCBI website (http://www.ncbi.nlm.nih.gov/BLAST) to align the obtained sequences. The Clustal X 1.82 software and MEGA 5.0 software were used to perform multiple comparison screening and construct the phylogenetic tree, and determine with which strain the ZWJ strain was clustered, and its homology was the highest.

### 4.3. Growth Curve of ZWJ Strain

#### 4.3.1. Fermentation

A single colony of the ZWJ strain was picked and inoculated into 100 mL of liquid medium, cultured with shaking at 180 rpm for 12 h at 37 °C (OD_600 nm_ = 0.8), and used as seed cultures. The seed cultures were added to thirteen flasks containing 250 mL of sterilized medium in a 2.0% (*v*/*v*) ratio, and fermented at 37 °C with shaking at 180 rpm for 33 h. Then, 30.0 mL of the fermentation broth in a bottle was centrifuged at 12,000× *g* for 5 min every 3 h, and the resulting supernatants were stored at 4 °C for subsequent use.

#### 4.3.2. Emulsification Index (E) of the Supernatant

The emulsification index, “E”, is a commonly used index for measuring the emulsification activity and emulsion stability of emulsifiers and is determined as follows. First, 2.0 mL of the above supernatant are added to a glass tube followed by the addition of 3.0 mL of vegetable oil, and mixing for 2 min (3000 rpm) using a vortex mixer to obtain the emulsion. Sterilized liquid medium was used as a blank control. The emulsion samples were placed at room temperature for 24 h and 168 h, respectively, and the emulsion layer height “he” and the total height of the mixture “ht” were substituted into Equation (1):

E_24_ or E_168_ = (he/ht) × 100 (1)

#### 4.3.3. Total Sugar Content of the Supernatant

The polysaccharide content is the difference between the total sugar content and the reducing sugar content. The total sugar content was determined by the phenol-sulfuric acid method [29]. First, the standard curve of the total sugar content was prepared by using glucose as the standard. The fermentation supernatant was added with phenol-sulfuric acid to determine the absorbance value A (at 490 nm) and the standard curve regression was established in advance. The equation (Y = 0.275 × −0.0041, *R*^2^ = 0.9991) gives the total sugar content of the fermentation broth. The content of reducing sugar was determined by the 3, 5-dinitrosalicylic acid (DNS) colorimetric method. Briefly, the supernatant of the fermentation was added to the DNS, and after boiling for 5 min, the absorbance value A was measured and substituted into the fermentation broth of the above standard curve regression equation to obtain the reducing sugar content. Data are given as the means ± SD, *n* = 3.

### 4.4. Preparation and Physicochemical Properties of EPS

The seed cultures, prepared as described in Section 4.3.1. above, was inoculated into a fermenter containing 2 L of medium. The fermentation conditions were as follows: agitation speed of 180 rpm, aeration rate of 200 L/h, temperature of 37 °C, and a total duration of 33 h. The cells were centrifuged at 12,000× *g* (4 °C, 5 min), and then filtered under pressure with a microporous membrane (0.45 μm). The filtered fermentation broth was concentrated by vacuum distillation at 60 °C. Then, 3 volumes of re-distilled absolute ethanol were added to the concentrate, and the mixture was thoroughly stirred and placed at 4 °C until a large amount of precipitate was formed at the bottom. The lower layer precipitate was collected by centrifugation (8000× *g*, 5 min), washed three times with re-distilled ethanol, and then completely dissolved in distilled water. The sample was next dialyzed for 2–3 days in the distilled water using a dialysis bag (3500 Da) and then freeze-dried. The lyophilized sample was re-dissolved in deionized water, and the protein was removed 3–5 times by the Sevage method to obtain a viscous liquid, which was again freeze-dried to obtain a crude product. The EPS was further separated and purified using a DEAE-52 cellulose column and a dextran G-75 column [15]. The obtained pure EPS was dissolved in deionized water, and the Mw was measured by gel permeation chromatography (GPC). The complete acid hydrolysate was analyzed for EPS monosaccharide composition by high performance liquid chromatography (HPLC) and nuclear magnetic resonance (NMR) spectroscopy analysis. Comparisons with glucose, glucuronic acid, arabinose, rhamnose, fructose, galactose, mannose, fucose and galacturonic acid (Sigma, ≥99%) (purification process and physical and chemical properties, etc.) are shown in the Supplementary Materials.

### 4.5. The Emulsion Behavior of EPS

First, according to the method described in Section 4.3.2 above, the E_24_ and E_168_ of the EPS at different concentrations (0.5%, 0.75%, 1.0%, 1.25% and 1.5%) is evaluated for different vegetable oils (sunflower oil, corn oil, soybean oil and olive oil as oil phase). The oil corresponding to larger E, smaller and more uniform droplets was selected as the most suitable oil phase. Then, a series of emulsions were prepared with the optimum oil and different concentrations of EPS, and the E value and droplet size were measured to select the most suitable emulsification concentration. Finally, a small amount of a stable emulsion layer (168 h) was applied and uniformly spread on a glass slide (no coverslip), and the droplet morphology was observed using an inverted microscope (Olympus CX31; Olympus Corp., Tokyo, Japan) with a 10× objective lens, and eventually photographed. In addition, the droplet size of each emulsion was measured using a static laser light scattering particle size analyzer (Mastersizer S particle size; Malvern Panalytical Ltd., Malvern, UK) and the equivalent diameter histogram was automatically plotted [30].

### 4.6. Preparation and Properties of the EPS/CPT Emulsion

First, the solubility of CPT in sunflower oil was determined. Then, 0.25 mg of CPT was dissolved in 3.0 mL of sunflower oil, used as the oil phase. Next, 0.15 g of EPS was dissolved in 10 mL of distilled water to prepare an aqueous phase. A 2.0 mL aqueous phase was placed in a glass test tube, and 3.0 mL of the oil phase was added and shaken for 10 min (3000 rpm) with a vortex mixer to obtain a milky white EPS/CPT emulsion (the final concentration of CPT was 0.005%, *w*/*w*). The type identification, viscosity, drug loading, spreadability and stability of the emulsion are described in the Supplementary Materials.

### 4.7. Drug Release In Vitro

The in vitro drug release by the EPS/CPT emulsion was determined according to the method described by Sonawane [21]. Under sink conditions, a dialysis bag (3500 Da) containing 10.0 g of the EPS/CPT emulsion was immersed in 20 mL of release medium (pH = 5.8 phosphate buffer saline (PBS) and methanol at a ratio of 7:3), placed in a water bath shaker at 37 °C and the release was conducted at 50 rpm. A volume of 200 µL was sampled every 1 h and an equal volume of isothermal release medium was added, and the cumulative release amount of the obtained sample was determined by HPLC analysis.

### 4.8. Ex Vivo Skin Permeability Test

#### 4.8.1. Skin Permeation

Transdermal permeation of CPT was measured using static Franz diffusion cells [4]. The upper epidermis of a fresh pig ear purchased from the slaughterhouse was depilated and washed, the fat layer was removed, and the remaining dermis and stratum corneum were washed with PBS, cut into cubes and fixed in the receiving chamber of the diffusion cell between the chambers, the stratum corneum was facing the drug delivery chamber (Effective diffusion area is 2.8 cm^2^). The drug was spread in the delivery chamber with the 0.5 mg free CPT group (CPT 0.005%, *w*/*w*) and the EPS/CPT emulsion group (CPT 0.005%, *w*/*w*), which was approximately 166.3 μg/cm^2^ (maximum skin tolerance according to CPT). The receiving chamber was PBS buffer (pH = 5.8, 6.5 mL), stirred at 37 °C, 300 rpm, and 250 µL of the receiving solution was taken at intervals, and an equal amount of PBS buffer was added. The receiving liquid taken at each time point was detected by HPLC analysis.

#### 4.8.2. Dermal Pharmacokinetic Study

Dermal pharmacokinetics was conducted by tape stripping according to the literature [4]. Skin tissue after completion of the above permeation experiment was removed from the diffusion cell and washed three times with PBS. The stratum corneum was removed by tape stripping (15 strips were stripped 15 times), and the cuticle-containing tape was cut into small pieces and extracted in 5 mL of methanol for 2 h, and then the supernatant was centrifuged. The remaining exfoliating skin (dermis layer) was cut into small pieces, homogenized, and the supernatant was centrifuged to determine the drug content.

### 4.9. In Vivo Animal Experiments

#### 4.9.1. Imiquimod Cream Induces Psoriasis Mouse Model

The establishment of a mouse psoriasis model using Imiquimod cream is recognized as one of the simple and easy in vivo methods to study psoriasis. In this study, 30 male specific pathogen-free (SPF) mice weighing 200 ± 10 g, were divided into a healthy control group, a model group, a free CPT group (oil phase as solvent), an EPS/CPT emulsion group and a positive control group (Commercially available preparation: Daivonex, CPT scalp solution). At the time of modeling, the mice were depilated on the back (2.0 cm × 3.0 cm), and 5.0% Imiquimod cream (200 μL) was applied evenly on the back and the right ear, once in the morning and evening for 8 d. The mice that successfully modeled the disease were started with treatment by the EPS/CPT emulsion and Daivonex on the 9th day, three times a day for 7 days.

#### 4.9.2. Evaluation of Indicators

During the course of the drug treatment, the surface morphology, scale thickness, erythema, and redness of each experimental area were visually observed and scored daily, using psoriasis-like lesion area and severity (erythema, scales, plaque, and hypertrophy). The psoriasis area and severity index (PASI) score was recorded in five levels of 0 points = none, 1 point = mild, 2 points = moderate, 3 points = severe, and 4 points = extremely severe. Each group of 6 mice was averaged.

#### 4.9.3. Histological Examination of the Skin and Spleen

After 24 h of the last administration, the mice from each group were sacrificed, and the spleen of each animal was dissected and the size was assessed. The skin tissue in the experimental area were fixed in 10% formalin, embedded in paraffin, sectioned (thickness about 0.5 mm), and stained with hematoxylin and eosin (H&E). Then, a photograph was taken. The histological scoring criteria were implemented in accordance with the Baker method and references.

#### 4.9.4. Analysis of the Serum Levels of the Cytokines TNF and IL6

Blood was collected from the eyelid of each mouse and allowed to coagulate for 30 min. Then, blood was centrifuge at 2500 rpm for 5 min at 25 °C, and serum was collected and stored at −80 °C. Eventually, IL6 and TNF levels were measured with the ELISA kits following the manufacturer’s instructions.

### 4.10. Skin Irritation Test

Twelve mice were depilated with an epilator and divided into four groups: saline, EPS/CPT emulsion, Daivonex and capsaicin. 200-mg sample above were evenly applied to the depilated area with a sterile cotton swab and held for 2 h. After 4 d of continuous administration, the skin’s redness and erythema were visually assessed to assess skin irritation as follows: No erythema (0), slight erythema or light pink (1), moderate erythema or dark pink (2), moderate to severe erythema or light red (3) and severe erythema or extreme redness (4). Three samples per group were averaged [4].

### 4.11. Data Analysis

The data were expressed as the $\bar{x}$ ± sd, and analyzed by the *t*-test with the blank group and analyzed using the Origin 8.1 software (OriginLab Corp., Northampton, MA, USA).

## 5. Conclusions

A strain of marine mangrove bacteria producing EPS was screened and identified. In view of the remarkable emulsifying activity of the newly identified EPS, an EPS/CPT emulsion was prepared by using it as an emulsifier, with sunflower oil as an oil phase, and CPT as the loaded drug. The emulsion was found to have moderate viscosity and excellent spreadability. The results of the in vitro drug release analysis revealed a certain sustained release effect. The in vitro skin drug penetration test showed that the emulsion enhances the penetration ability of CPT and increases its accumulation in the dermis. Animal experiments confirmed that the emulsion enhances the effect of CPT in the treatment of psoriasis and also reduced the skin irritation. Compared with systemic drug administration, this method improved the safety. In conclusion, for the first time, in this study, EPS was used as a moisturizer and emulsifier in the treatment of psoriasis vulgaris, and the findings lay a theoretical foundation for the application of EPS as medical materials. In the future, we plan to expand the research on the application of EPS in the treatment of other dermatological diseases.

## Figures and Tables

**Table 1:** Emulsion type, viscosity, spreading value, average particle size and drug content of prepared exopolysaccharides/calcipotriol (EPS/CPT) emulsion.

Emulsion Type	Viscosity (Torque Value Is 50%)	Spreading Value (20 μL Sample)	Average Droplet Size	Calcipotriol (CPT) Content %
Oil in Water (O/W)	105.3 ± 6.8 mPa·s	542 ± 30 mm^2^	2433 nm	0.00492

**Table 2:** Psoriasis area and severity index (PASI) score sheet.

Groups	Days						
	1	2	3	4	5	6	7
Healthy control	0 ± 0.00	0 ± 0.00	0 ± 0.00	0 ± 0.00	0 ± 0.00	0 ± 0.00	0 ± 0.00
Model Group	4.00 ± 0.00	4.00 ± 0.00	4.00 ± 0.00	3.33 ± 0.47	3.17 ± 0.37	3.00 ± 0.00	3.00 ± 0.00
EPS/CPT emulsion	3.33 ± 0.47	3.00 ± 0.00	2.83 ± 0.37	* 2.00 ± 0.00	* 1.67 ± 0.47	* 1.50 ± 0.50	** 1.00 ± 0.00
Free CPT	4.00 ± 0.00	3.33 ± 0.47	3.17 ± 0.37	3.00 ±0.00	2.83 ± 0.37	* 2.50 ± 0.50	* 2.00 ± 0.00
Positive control group	3.33 ± 0.47	3.00 ± 0.00	2.67 ± 0.37	* 2.00 ± 0.00	* 2.00 ± 0.00	* 1.67 ± 0.47	* 1.67 ± 0.37

Data are expressed as mean ± SD (*n* = 6), (* *p* < 0.05, ** *p* < 0.01).

**Table 3:** Severity of the skin irritation scores for all the animals in each group.

Groups	Days			
	1	2	3	4
Saline	0 ± 0.00	0 ± 0.00	0 ± 0.00	0 ± 0.00
EPS/CPT emulsion	0 ± 0.00	0 ± 0.00	0 ± 0.00	* 0.7 ± 0.47
Daivonex	0 ± 0.00	0.33 ± 0.47	0.67 ± 0.47	1.0 ± 0.00
Capsaicin	3.0 ± 0.00	3.0 ± 0.00	3.33 ± 0.47	3.33 ± 0.47

Data are expressed as mean ± SD (*n* = 3). * *p* < 0.05.

## Supplementary Materials

Supplementary Materials can be found at https://www.mdpi.com/1422-0067/21/1/77/s1.
