# Supplementary material for: Preparation of Calcipotriol Emulsion Using Bacterial Exopolysaccharides as Emulsifier for Percutaneous Treatment of Psoriasis Vulgaris
Source: Int J Mol Sci. 2019 Dec 20;21(1):77. doi: 10.3390/ijms21010077 (PMC6982318; doi:10.3390/ijms21010077)
Supplement: Supplementary file 1 [file ijms-21-00077-s001.pdf]

International Journal of Molecular Sciences
MDPI

Supplementary Information

S1.1. Preparation and Physicochemical Properties of EPS

S1.1.1. Isolation and Purification of EPS

The crude EPS was further separated and purified using a DEAE-52 cellulose column and a dextran G-75 column, using a 0–2 M NaCl solution as an eluent. The polysaccharide content (490 nm) was determined by phenol-sulfuric acid method, and a separation curve was drawn. Finally, the components were combined and dialyzed (3500 Da), followed by lyophilization, in order to obtain a white loose pure EPS.

S1.1.2. Gel Permeation Chromatography (GPC) for EPS

We took 8.0 mg sample, added 3.0 mL of water to dissolve and filtered with 0.22 μm aqueous filter. Under the solvent system of water phase, GPC analysis of EPS was carried out to obtain the average molecular weight and distribution information of samples. The GPC system consisted of a PL aquagel-OH MIXED-H 8 μm, 300 × 7.5 mm, and connected to a PL aquagel-OH 8 μm, 50 × 7.5 mm column. The column temperature was 40 °C, loading volume was 50 μL and flow rate was 0.5 mL/min.

S1.1.3. Monosaccharide Composition (HPLC and NMR Analysis)

The hydrolyzed mixed monosaccharide sample was dissolved in D_2O and analyzed by a nuclear magnetic resonance apparatus (Bruker, 500 M·Hz).

S1.1.4. Moisturizing Effect of EPS

According to the "tea bag" method reported by Zhu et al. (2018) [1], the obtained data were substituted into formulas (1) and (2) to calculate the liquid absorption ratio and water retention rate of EPS.

$$\text{Liquid absorption ratio (Q) (g/g)} = (Wt - W1 - W0) / W0 \quad (1)$$

Among them, W0: EPS mass (g) when dry; W1: wet tea bag mass (g); Wt: total mass (g) of EPS after absorbing liquid and tea bag after achieving swelling balance. All measurements were performed in triplicate.

$$\text{Liquid retention rate R\%} = W3/W2 \times 100\% \quad (2)$$

where W2: initial mass (g) of EPS and tea bag after liquid absorption; W3: total weight (g) of water-absorbing EPS and tea bag after being placed for different time. Data are given as means ± SD, *n* = 3.

S1.1.5. Rheological Property of EPS

EPS (0.2 g) was weighed and dissolved in 20 mL of deionized water to a final concentration of 1.0% (*w*/*v*) and placed at 25 °C for 2 h. The shear stress and viscosity of the EPS solution were measured by a rheometer. The rotor was selected to be PP50, and the shear rate was between 0.1–100 s^{-1}. The data were plotted with Origin 9.0 and plotted.

S1.2. Physicochemical Characteristics of EPS/CPT Emulsion

S1.2.1. Characteristic Solubility of CPT in Sunflower Oil

First, 0.01, 0.02, 0.04, 0.06, 0.08 and 0.10 mg/mL of CPT sunflower oil solution were prepared, and the peak area was determined by HPLC to establish a standard curve: Y = 0.004491 X + 0.0003011 (r^2 = 0.9981). Then, 0.3 mg of CPT was added to 3.0 mL of sunflower oil, mixed in a vortex mixer for 10 min, heated at 40 °C for 5 min, and shaken at 37 °C for 50 h in a 50 rpm water bath. After centrifugation at 12,000 rpm for 10 min, the supernatant was filtered through a 0.45 μm lipophilic microporous membrane, and 10 μL of the supernatant was diluted with 20 μL of ethyl acetate and 1 mL of methanol. After HPLC analysis, the values were substituted into the above standard curve to obtain the solubility of CPT in sunflower oil. The HPLC conditions were: C18 column (4.6 × 150 mm ID, droplet size 5 μm), mobile phase Acetonitrile: water: methanol (45: 25: 30, *v*/*v*), flow rate 1.0 mL/min, column temperature 40 °C, injection volume 20 μL.

S1.2.2. Identification of Emulsion type

Emulsion that can uniformly disperse Sudan red is W/O type, and that can uniformly disperse methylene blue is O/W type.

S1.2.3. Droplet Size Distribution

A small amount of the emulsion was uniformly applied to a glass slide (no coverslip), and the morphology of the emulsion droplets was observed under an inverted microscope (10× objective lens). The droplet size of each emulsion was measured by Image J software, and an equivalent diameter histogram was automatically drawn.

S1.2.4. Viscosity Detection of EPS/CPT Emulsion

The dynamic viscosity of the EPS/CPT emulsion was measured by a Brookfield DV-III viscometer (LV3 rotor). 20 mL emulsion was added into the sample cup, the speed and the torque value were set as 20 rpm and 20–80%, respectively. Each sample was detected three times to get the average value (system temperature 25 °C).

S1.2.5. Drug Loading

Due to the extremely low CPT content, the HPLC method was used to determine the drug content of the emulsion. We weighed accurately 1.0 g of EPS/CPT emulsion into a brown volumetric flask and added the appropriate amount of methanol. We then heat the vortex at 40 °C to completely dissolve the CPT, added methanol to volume, and shook well. Centrifuged at 10,000 rpm for 10 min, a small amount of the solution was filtered through a 0.45 μm microporous membrane, and 10 μL was diluted with 20 μL of ethyl acetate and 1 mL of methanol. According to the HPLC analysis in the "*S1.2.1.*" method, the peak area value was substituted into the standard curve in "*S1.2.1.*", and the CPT content (%) was calculated. The HPLC conditions were the same as "*S1.2.1.*"

S2.2.6. Spreadability

The spreadability of EPS/CPT emulsion (20 μL) was conducted by a glass plate mold with a central circular orifice and the change in spreading area was observed as a function of change in weight [2].

S1.2.7. Stability of EPS/CPT Emulsion

The EPS/CPT emulsion was stored at 4 °C and 25 °C for 1 month, and the droplet size change, aggregation and stratification were examined every 5 days [3].

S2. Results

S2.1. Preparation and Physical–Chemical Properties of EPS

S2.1.1. Isolation and Purification of EPS

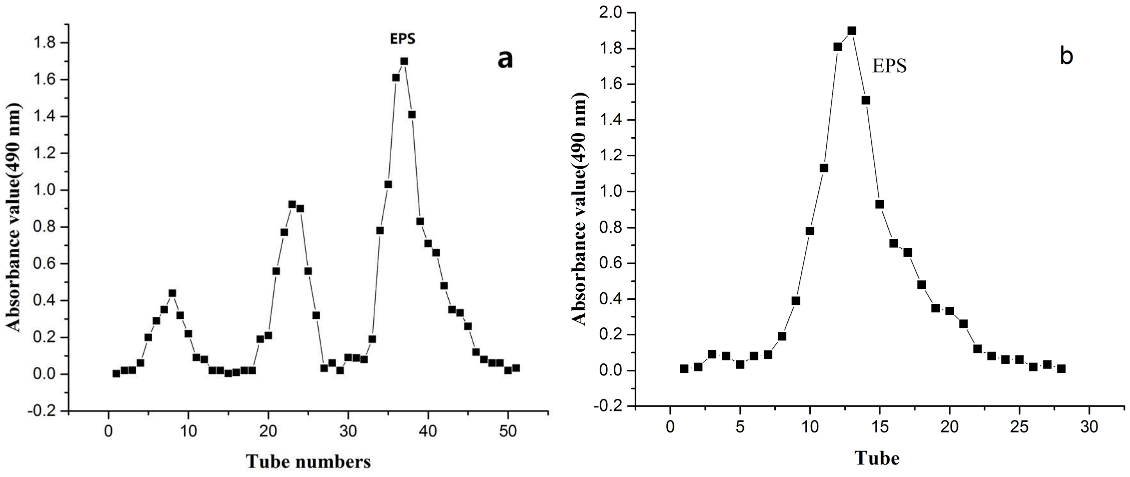

Figure S1. (**a**) DEAE-52 cellulose column elution curve for crude EPS; (**b**) Sephadex G-75 column elution curve.

S2.1.2. GPC of EPS

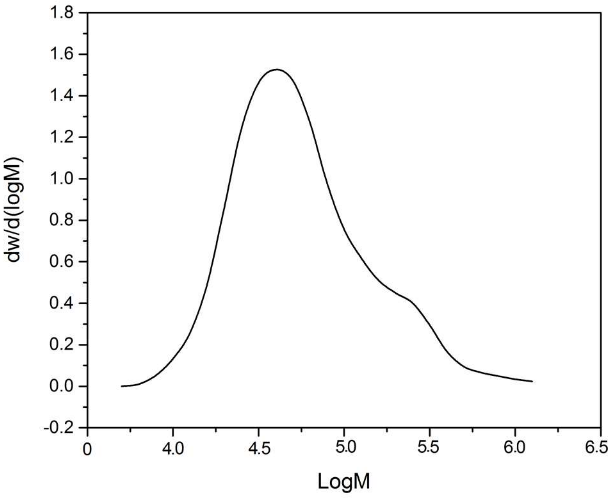

Figure S2. Molecular weight distribution of EPS from gel permeation chromatography (GPC) detection report. (X-axis: the logarithm of molecular weight; Y-axis: The Y-axis value of a point on the curve is the related value of the content of the sample molecules at each molecular weight.).

Table S1. EPS molecular weight calculation results.

Mn	Mw	Mz	Mw/Mn
1.89×10^5	2.61×10^5*	4.72×10^5	1.38

* Generally refer to Mw (the weight average molecular weight).

S2.1.3. HPLC and NMR Analysis of EPS

Table S2. Standard curve of mixed monosaccharide standard solution.

Monosaccharides	Retention Time	Equation of Standard Curve	Residual Standard Error
Mannose	12.134	y = 84.22247x-38.15190	0.99817
Rhamnose	14.101	y =125.26783x-88.70865	0.99593
Glucuronic acid	17.648	y = 84.11379x-49.92372	0.99622
Xylose	19.193	y = 103.07485x-28.73048	0.99863
Galacturonic acid	19.845	y = 92.92633x-39.82440	0.99741
Glucose	23.697	y = 82.65695x-31.05636	0.99806
Galactose	27.663	y = 49.55186x-35.47082	0.99612
Arabinose	31.555	y = 114.43241x-27.66505	0.99647
Fucose	37.896	y = 76.63759x-44.26851	0.99615

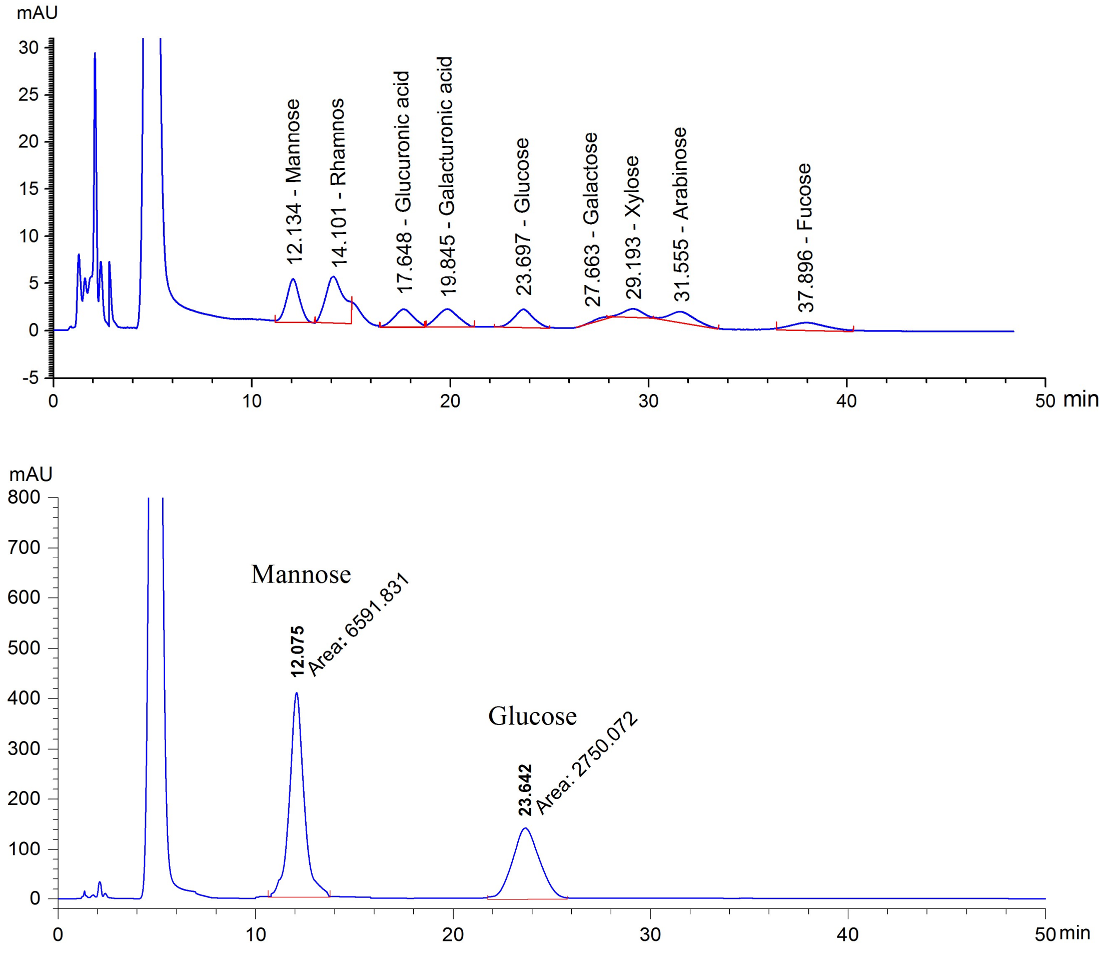

Figure S3. **(a)** Mixed monosaccharide standards solution detection curve; (**b**) The monosaccharide composition detection curve of EPS.

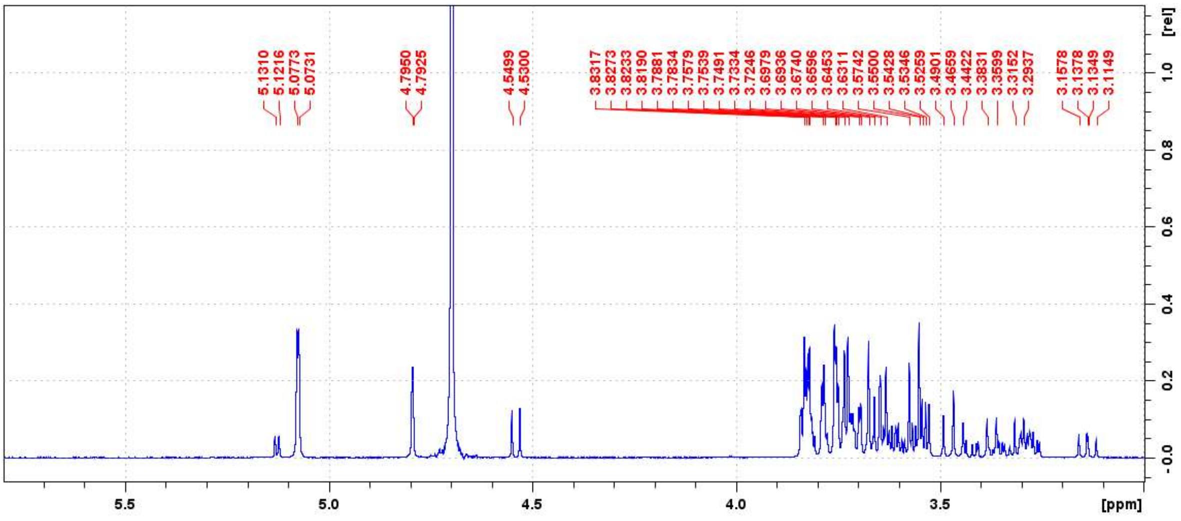

Figure S4. ^{1}H-NMR of EPS.

^{1}H-NMR peak assignment: The ^{1}H-NMR signal of the EPS hydrolyzed monosaccharide was concentrated between 3.00–5.50, and was mainly divided into two regions: an anomeric hydrogen region (5.80–4.50) and a cyclic proton region (4.00 to 3.00). The signal in the anomeric region is clear: δ 5.13–5.12 and δ 4.55–4.53 are the chemical shifts of the glucose sugar end stromate; δ 5.08 and δ 4.79 are the end stromate peaks of mannose. However, the cyclic proton region signal is very complex: δ 3.80, δ 3.77, δ 3.63–3.60, δ 3.44–3.40, δ 3.38–3.36, δ 3.31–3.29 and δ 3.16–3.11 series of peaks may be due to the chemical shift of cyclic proton region of glucose; δ 3.83–3.82, δ 3.79–3.73, δ 3.68–3.63, δ 3.58–3.5, δ 3.49–3.44 and δ 3.30–3.27 may correspond to the cyclic proton region of mannose.

Table S3. The monosaccharide composition of EPS.

**Samples**	**Monosaccharide Ratio (%)**	
	**Mannose**	**Glucose**
EPS	70.56 %	29.44%

S2.1.4. Liquid Retention Performance

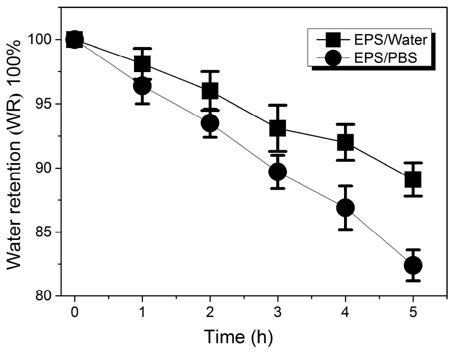

Figure S5. The water retention (WR%) capacity of EPS.

S2.1.5. Rheological Property of EPS

The rheological characteristics show that the apparent viscosity of the EPS becomes smaller as the shear force increases (Figure S6), which is characterized by shear thinning and is a pseudoplastic fluid characteristic in the classical non-Newtonian fluid.

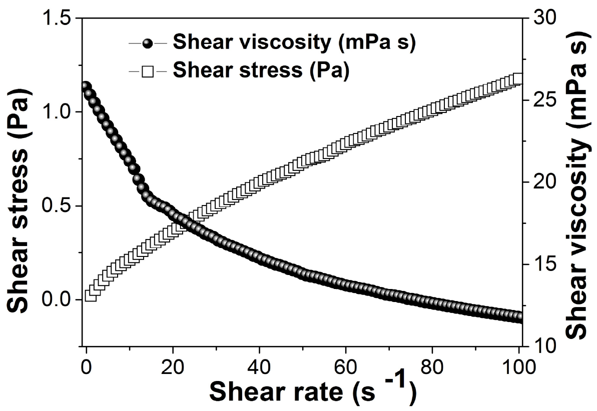

Figure S6. Viscosity-shear rate profile (c) of EPS (1.0%).

S2.1.6. Emulsion Prepared with Different Oils and Emulsification Index

Table S4. Emulsification indices (E_{24} and E_{168}) for emulsions prepared with different oils with EPS and xanthan gum (both at 1.5%). E24 means emulsification index after 24 h; E168 means emulsification index after 168 h. Data are given as the means ± SD, $n = 3$. (* $p < 0.05$).

Oils	Type of gum or exopolysaccharide					
	EPS		Xanthan gum		Water	
	E24	E168	E24	E168	E24	E168
Olive oil	76.7 ± 0.9	65.2 ± 1.9	48.6 ± 1.1	44.8 ± 1.2	8.5 ± 1.4	7.6 ± 0.9
Sunflower oil	96.2 ± 0.7 *	82.5 ± 0.8 *	49.1 ± 1.4	45.3 ± 2.5	9.4 ± 0.3	8.7 ± 0.4
Corn oil	69.6 ± 0.9	56.8 ± 0.9	47.5 ± 0.8	45.4 ± 1.3	8.3 ± 0.9	7.6 ± 1.2
Soybean oil	66.0 ± 1.7	52.1 ± 1.3	43.0 ± 1.3	41.4 ± 1.2	7.7 ± 1.1	6.7 ± 0.2

S2.2. Characterization of Physical and Chemical Properties of EPS/CPT

S2.2.1. Solubility of CPT in Sunflower Oil

The solubility of CPT in sunflower oil was >0.1 mg/mL. The results indicate that 0.25 mg of CPT is completely soluble in 3.0 mL of sunflower oil when preparing EPS/CPT emulsion (0.005%, *w*/*w*).

S2.2.2. Identification of Emulsion Type

After identification, the EPS/CPT emulsion was O/W type.

S2.2.3. Droplet Size of EPS/CPT Emulsion

The average droplet size of EPS/CPT emulsion is 2433 nm (Figure S7). Since the content of CPT is extremely small, the effect on the droplet size of the emulsion is extremely small.

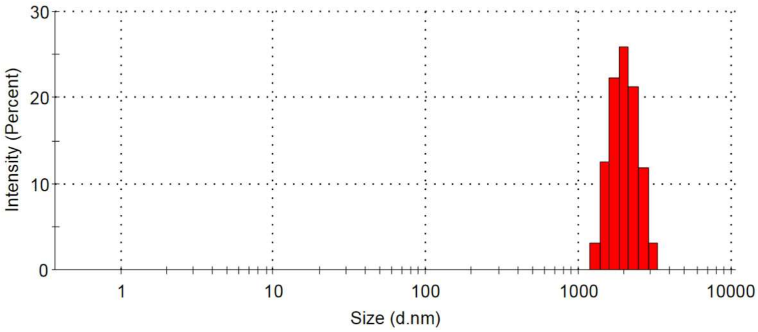

Figure S7. Size distribution of EPS/CPT emulsion (Average droplet size: 2433 nm).

S2.2.4. Viscosity Detection of EPS/CPT Emulsion

It was tested that the dynamic viscosity of the EPS/CPT emulsion was 105.3 ± 6.8 mPa·s at a torque value of 50%.

S2.2.5. Drug Loading

The EPS/CPT emulsion drug content was 0.00495%, which was 99.2% of the labeled amount.

S2.2.6. Spreadability

Spreadability profiles of EPS/CPT emulsion and free CPT are shown in Figure S8. The spreading values of the EPS/CPT emulsion increased with increase in the weight, which indicates the spreadability of EPS/CPT emulsion can be enhanced by rubbing the emulsion onto the skin.

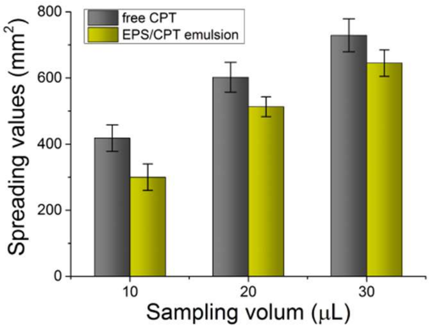

Figure S8. Spreadability values of EPS/CPT emulsion and free CPT at different volumes (10, 20 and 30 μL). Data presented as means ± SD (n = 3).

S2.2.7. Stability of EPS/CPT Emulsion

The EPS/CPT emulsion was allowed to stand at 4 °C and 25 °C for 30 d, and the droplet size was observed and measured every 5 d. The results, as shown in Figure S9, although the droplet size increased slowly, it remained white milky appearance without delamination and aggregation, indicating that the EPS/CPT emulsion has high storage stability. The stability at 4 °C was higher than 25 °C.

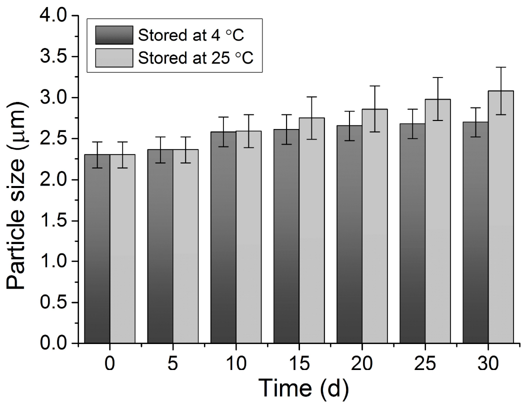

Figure S9. Droplet size changes of the EPS/CPT emulsion stored at different temperatures for 30 d. (X ± SD, *n* = 3).

References

1. Zhu, W.J.; Wang, Y.Z.; Yan, F.; Song, R.T.; Li, Z.J.; Li, Y.Q.; Song, B. Physical and chemical properties, percutaneous absorption-promoting effects of exopolysaccharide produced by *Bacillus atrophaeus* WYZ strain. *Carbohydr. Polymers* **2018**, *192*, 52–60.
2. Daudt, R.M.; Back, P.I.; Cardozo, N.S.M.; Marczak, L.D.F.; Külkamp-Guerreiro. I.C. Pinhão starch and coat extract as new natural cosmetic ingredients: topical formulation stability and sensory analysis. *Carbohydr. Polymers* **2015**, *134*, 573–580.
3. Kaci, M.; Arab-Tehrany, E.; Desjardins, I.; Banon-Desobry, S.; Desobry, S. Emulsifier free emulsion: Comparative study between a new high frequency ultrasound process and standard emulsification processes. *J. Food Eng.* **2016**, *194*, 109–118.

© 2019 by the authors. Licensee MDPI, Basel, Switzerland. This article is an open access article distributed under the terms and conditions of the Creative Commons Attribution (CC BY) license (http://creativecommons.org/licenses/by/4.0/).
